# Low-level mitochondrial heteroplasmy modulates DNA replication, glucose metabolism and lifespan in mice

**DOI:** 10.1038/s41598-018-24290-6

**Published:** 2018-04-12

**Authors:** Misa Hirose, Paul Schilf, Yask Gupta, Kim Zarse, Axel Künstner, Anke Fähnrich, Hauke Busch, Junping Yin, Marvin N. Wright, Andreas Ziegler, Marie Vallier, Meriem Belheouane, John F Baines, Diethard Tautz, Kornelia Johann, Rebecca Oelkrug, Jens Mittag, Hendrik Lehnert, Alaa Othman, Olaf Jöhren, Markus Schwaninger, Cornelia Prehn, Jerzy Adamski, Kensuke Shima, Jan Rupp, Robert Häsler, Georg Fuellen, Rüdiger Köhling, Michael Ristow, Saleh M. Ibrahim

**Affiliations:** 1 Lübeck Institute of Experimental Dermatology, University of Lübeck, Lübeck, Germany; 2 Energy Metabolism Laboratory, Institute of Translational Medicine, Swiss Federal Institute of Technology (ETH) Zurich, Schwerzenbach, Switzerland; 3 Medical Systems Biology Group, Lübeck Institute of Experimental Dermatology, University of Lübeck, Lübeck, Germany; 4 Institute for Cardiogenetics, University of Lübeck, Lübeck, Germany; 5 Institute of Medical Biometry and Statistics, University of Lübeck, University Medical Center Schleswig-Holstein, Campus Lübeck, Lübeck, Germany; 6 StatSol, Lübeck, Germany; 7 Max Planck Institute for Evolutionary Biology, Evolutionary Genomics, Plön, Germany; 8 Institute for Experimental Medicine, Section of Evolutionary Medicine, Christian-Albrechts-University of Kiel, Kiel, Germany; 9 Center of Brain Behavior & Metabolism, Molecular Endocrinology, University of Lübeck, Lübeck, Germany; 10 Center of Brain Behavior & Metabolism, Clinical Endocrinology and Metabolism, University of Lübeck, Lübeck, Germany; 11 Institute for Experimental and Clinical Pharmacology and Toxicology, University of Lübeck, Lübeck, Germany; 12 Center of Brain, Behavior & Metabolism, University of Lübeck, Lübeck, Germany; 13 Helmholtz Center, German Research Center for Environmental Health, Institute of Experimental Genetics, Genome Analysis Center, Neuherberg, Germany; 14 Department of Infectious Disease and Microbiology, University of Lübeck, Lübeck, Germany; 15 Institute of Clinical Molecular Biology, Christian-Albrechts-University Kiel, Kiel, Germany; 16 Institute for Biostatistics and Informatics in Medicine and Ageing Research, Rostock University Medical Center, Rostock, Germany; 17 Oscar-Langendorff-Institute of Physiology, Rostock University Medical Center, Rostock University, Rostock, Germany; 18 College of Medicine and Sharjah Institute for Medical Research, University of Sharjah, Sharjah, United Arab Emirates; 19 Present Address: Leibniz Institute for Prevention Research and Epidemiology, BIPS GmbH, Department Biometry and Data Management, Unit Statistical Methods in Genetics and Live-Course Epidemiology, Bremen, Germany

## Abstract

Mutations in mitochondrial DNA (mtDNA) lead to heteroplasmy, i.e., the intracellular coexistence of wild-type and mutant mtDNA strands, which impact a wide spectrum of diseases but also physiological processes, including endurance exercise performance in athletes. However, the phenotypic consequences of limited levels of naturally arising heteroplasmy have not been experimentally studied to date. We hence generated a conplastic mouse strain carrying the mitochondrial genome of an *AKR/J* mouse strain (B6-mt^AKR^) in a *C57BL/6 J* nuclear genomic background, leading to >20% heteroplasmy in the origin of light-strand DNA replication (OriL). These conplastic mice demonstrate a shorter lifespan as well as dysregulation of multiple metabolic pathways, culminating in impaired glucose metabolism, compared to that of wild-type *C57BL/6 J* mice carrying lower levels of heteroplasmy. Our results indicate that physiologically relevant differences in mtDNA heteroplasmy levels at a single, functionally important site impair the metabolic health and lifespan in mice.

## Introduction

Mitochondria play a critical role in maintaining cellular activities by generating energy in the form of adenosine triphosphate (ATP)^1^. Additionally, mitochondria function as a signaling platform, e.g., mitochondrial reactive oxygen species control a wide range of biological processes, including epigenetics, autophagy, and immune responses^2^. Since mitochondria are involved in such critical cellular activities, mitochondrial dysfunction has been linked to various degenerative and metabolic conditions (e.g., Alzheimer’s disease and diabetes), cancer, and aging in humans, as has been supported by experimental evidence^3–6^.

Mitochondria carry their own mitochondrial DNA (mtDNA), which encodes codes 13 OXPHOS complex genes, two ribosomal RNA genes, and 22 transfer RNA genes^7^. The mode of inheritance of mtDNA is maternal, and hundreds to thousands of mtDNA copies exist in a cell^1^. Mutations in mtDNA have been categorized into three groups: deleterious mutations, somatic mutations and adaptive polymorphisms^1^. Deleterious mutations result in severe mitochondrial dysfunction and are causal for maternally inherited mitochondrial disease such as Leber’s hereditary optic neuropathy (LHON)^8^ and mitochondrial encephalomyopathy, lactic acidosis, and stroke-like episodes (MELAS)^9^. Somatic mtDNA mutations accumulate within various tissues with age, and it has been experimentally shown that increased somatic mtDNA mutations exhibit aging phenotypes in mice^10,11^. In contrast, adaptive polymorphisms may be associated with survival under different climatic conditions or nutritional availability^1^.

Conplastic mouse strains are a unique and powerful tool to investigate the impact of mutations in mtDNA under a wide spectrum of physiological and pathological alterations^12^, including aging^13–15^. Of note, the study by Kauppila *et al*. investigating the impact of heteroplasmy on lifespan reported that higher levels of maternally inherited *de novo* mutations/heteroplasmy lead to severe pathological consequences^13^. Higher levels of mutations/heteroplasmy rarely occur naturally, apart from cancers^16,17^. In contrast, lower levels of maternally inherited heteroplasmy commonly exist^18,19^, while their phenotypic consequences have not been experimentally studied to date.

We previously generated a series of conplastic mouse strains, which carry distinct and stable mutations over generations in mtDNA on a *C57BL/6 J* nuclear genomic background^20^ and provide a unique opportunity to study the impact of natural variation of mtDNA on various biological and pathological processes. Since the mtDNA of those strains was previously sequenced using Sanger sequencing, which did not allow us to accurately determine their levels of heteroplasmy, we here performed next-generation sequencing of the mtDNA of all of our previously constructed conplastic strains and discovered a stable, maternally inherited, and low-level heteroplasmic mutation at nt5172 in the origin of L-strand replication (Supplementary Table S1). Moreover, the levels of the heteroplasmy varied between the *C57BL/6 J* and *C57BL/6J-mt*^*AKR/J*^ strains. Using this unique resource, we studied the impact of natural low-level heteroplasmy on aging, and demonstrated its consequences, including an impact on mtDNA copy number ratio and the regulation of metabolic processes, which may be causative for a shorter lifespan.

## Results

### Deep-sequencing of mtDNA prepared from B6-mt^AKR^ reveals the presence of low levels of a heteroplasmic mutation at position 5172 in the OriL

**Figure 1:**
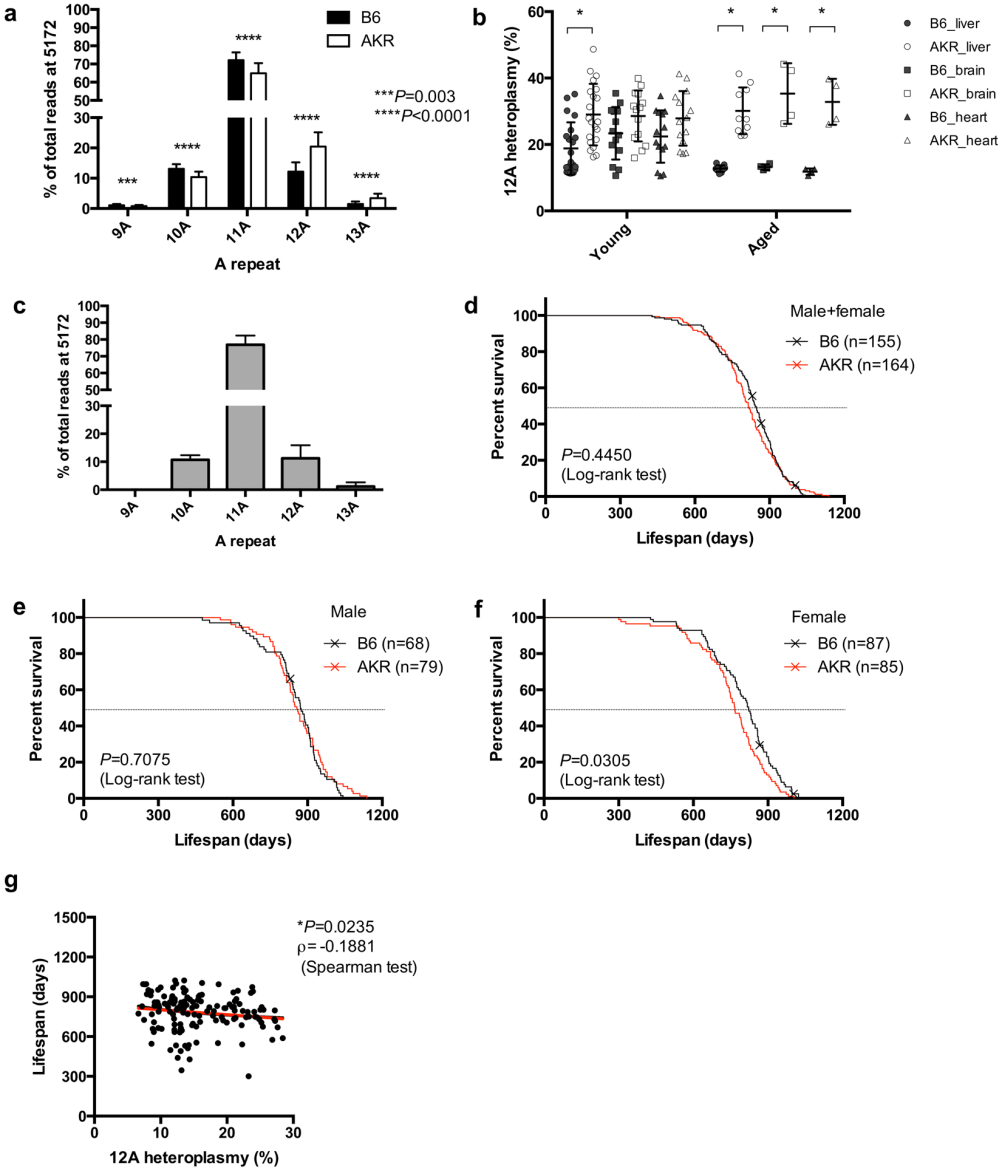
Levels of 12 A heteroplasmy at 5172 in the origin of light strand replication negatively correlate to lifespan in mice. **(a)** Next generation sequencing of blood DNA obtained from moribund B6 and B6-mt^AKR^ (AKR) mice shows that AKR exhibits significantly higher levels of the 12 A heteroplasmy at 5172 in the origin of light strand replication (OriL) compared with B6 (***P = 0.003, ****P < 0.0001; two-way ANOVA). **(b)** The higher levels of 12 A heteroplasmy in OriL are observed in different tissues (liver, brain and heart). The difference becomes more prominent when mice are aged (17–22 months old). *P < 0.05, multiple *t-*test. **(c**) The heteroplasmy at 5172 is also detected in wild house mice (*Mus musculus domesticus*) caught on farms (n = 215). **(d**–**f)** Survival curve of B6 and B6-mt^AKR^ (AKR) in both sexes (**d**), males (**e**), and females (**f**). Female AKR display a significantly shorter lifespan compared with B6 (*P* = *0*.*0305*, log-rank test). **(g)** The higher 12 A heteroplasmy levels in OriL correlate with the shorter lifespan (ρ = −0.1881 *P* = 0.0235, Spearman test).

First, we deep-sequenced the mtDNA of a series of conplastic strains that we previously generated^20^, and identified a strain carrying low levels of heteroplasmy at position 5172 in OriL (Supplementary Table S1). This particular strain carries the mtDNA of *AKR/J* (*C57BL/6J-mt*^*AKR/J*^; B6-mt^AKR^) on a *C57BL/6 J* (B6) background. Consistent with previously published data^21,22^, the adenine-repeat number varies among individuals at this position. Specifically, the majority of mtDNA (approximately 60–70%) carries eleven adenines (“11 A”), while the remaining percentage exhibits either 9, 10 or more than 11 adenines (9 A, 10 A, 12 A, 13 A). B6-mt^AKR^ mice have higher levels of >11 A heteroplasmy compared to B6 (Fig. 1a, 12 A and 13 A; *P* < *0*.*0001* for both, two-way ANOVA). While the levels of the 12 A heteroplasmy at OriL vary individually, overall B6-mt^AKR^ exhibits >20% heteroplasmy in comparison to B6, which carries approximately 10% heteroplasmy at the same position in different organs and at different ages (Fig. 1b). Interestingly, the heteroplasmy at 5172 is also observed in free-living house mice (*Mus musculus domesticus*) caught on farms (n = 215, Fig. 1c), and the pattern of heteroplasmy resembles that of B6 (Fig. 1a). This indicates that the patterns observed in inbred laboratory mice are also present in nature, where they may represent a consequence of mutation pressure and possibly be subject to natural selection.

### Levels of 12 A heteroplasmy at 5172 in the OriL negatively correlate with lifespan

To investigate the impact of heteroplasmy at the OriL on murine lifespan and aging-related phenotypes, we conducted a longevity study using a large cohort of this strain, and B6 control mice. Female B6-mt^AKR^ live significantly shorter than B6 mice (approx. 50 days, *P* = *0*.*0305*, log-rank test), while the lifespan of males does not differ between strains (Fig. 1d–f, Supplementary Tables S1a–d, and Supplementary Fig. S1a–c). Neither the aging score (i.e., the presence of alopecia, graying hair, body mass reduction, and kyphosis, as previously described^23^), the tumor incidence, nor the incidence of ulcerative dermatitis is significantly different between the strains (Supplementary Fig. S1d–f). Sequencing of genomic DNA derived from blood obtained from moribund female mice (B6; n = 84, B6-mt^AKR^; n = 61) reveals an inverse correlation between lifespan and higher levels of heteroplasmy (Fig. 1g, ρ = −0.1881, *P* = 0.0235, Spearman test).

### Higher 12 A heteroplasmy at 5172 in the OriL reduces the mtDNA/nDNA ratio, while the expression level of the mtDNA-encoded genes is increased

**Figure 2:**
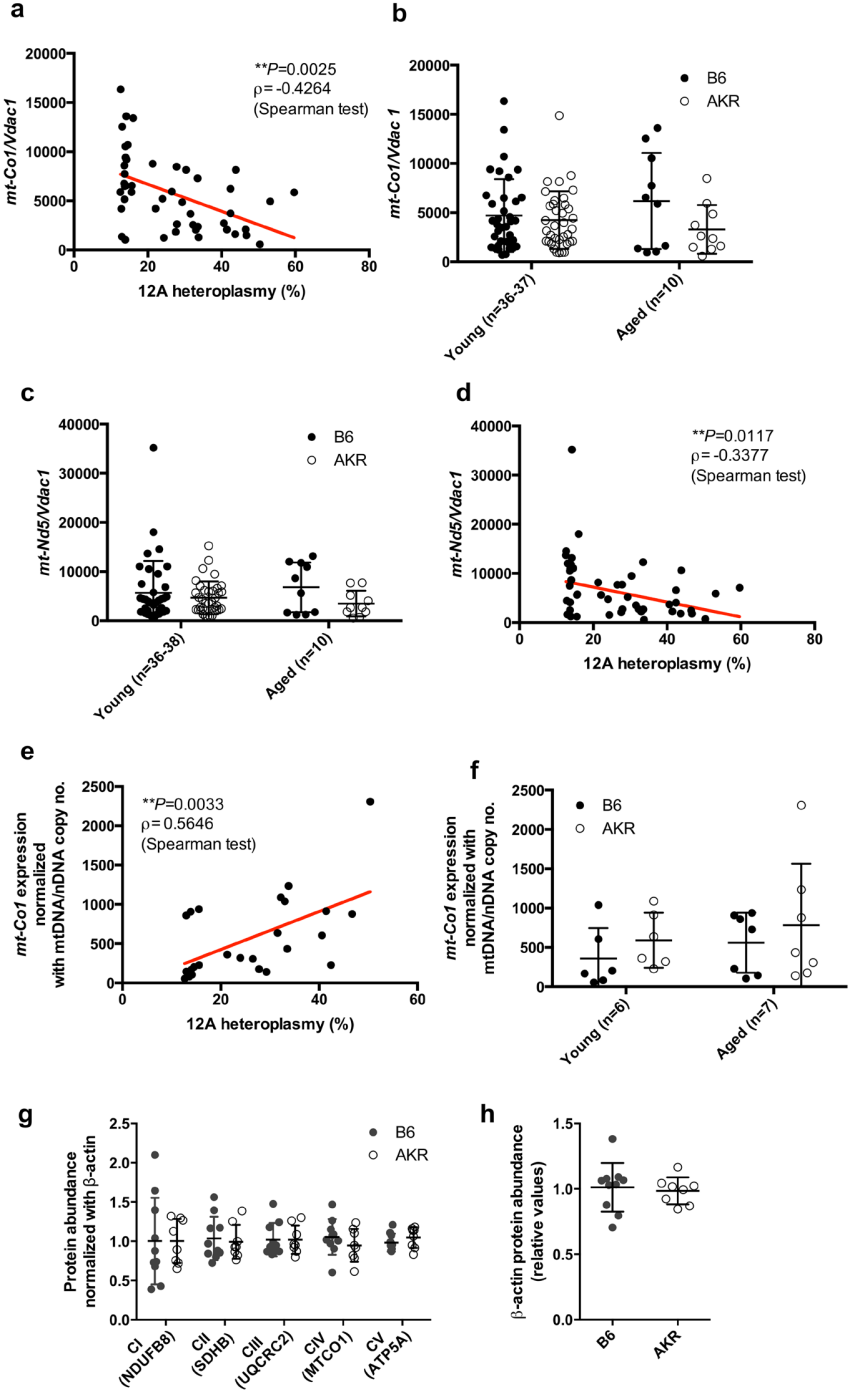
Higher levels of 12 A heteroplasmy at 5172 in the origin of light strand replication reduces mtDNA/nDNA copy number ratio, while the expression levels of the mtDNA-encoded gene are increased. **(a)** The ratio of mtDNA (*mt-Co1*)- to nDNA (*Vdac1*) copy number was determined using liver genomic DNA obtained from B6 and B6-mt^AKR^ (AKR) mice (females, young: 3 months of age, aged: 18–22 months of age). The copy number ratio (*mt-Co1/Vdac1*) negatively correlates to levels of 12 A heteroplasmy in OriL. N = 48, *ρ* = −0.4264, *P = 0.0025, Spearman test. **(b)** The values presented in **a**. were compared between strains, for which no difference is observed between B6 and AKR in each age group. **(c)** MtDNA gene copy number ratio based on *mt-Nd5/Vdac1* shows the same trend found by *mt-Co1/Vdac1*. **(d)** Higher 12 A heteroplasmy levels in OriL also correlate with the copy number ratio of *mt-Nd5/Vdac1*. **(e)** The expression levels of *mt-Co1* in liver mitochondrial RNA were quantified using droplet digital PCR. The *mt-Co1* expression normalized to the *mt-Co1* copy number ratio reveals a significant correlation with the levels of 12 A heteroplasmy. ρ = 0.5646, **P = 0.0033, Spearman test. **(f)** The values presented in **e**. display no significant difference when compared between the strains in each age group. **(g)** Quantified values of Western blotting of liver samples reveal unaltered protein levels of mitochondrial OXPHOS subunits. Females, three months of age, n = 10 (B6), n = 8 (AKR). **(h)** The same samples tested in **g**. were quantified for beta-actin.

Next, we aimed to determine the functional consequence(s) of 12 A heteroplasmy. Since the OriL is essential for mtDNA replication^22^, the ratio of mtDNA to nuclear genome copies was investigated by quantifying *mt-Co1* and *Vdac1* copies, respectively. We observe an inverse correlation with levels of 12 A heteroplasmy (*ρ* = −0.4264, *P* = 0.0025, Spearman test, Fig. 2a), while the overall ratio is unaltered between B6-mt^AKR^ and B6 at different ages (Fig. 2b). This is likely due to the high variability of 12 A heteroplasmy in individual mice within each strain. This 12A-correlated phenomenon is also observed for another mitochondrially encoded gene, *mt-Nd5*, again normalized to nuclear *Vdac1* (Fig. 2c,d). Thus, higher levels of 12 A heteroplasmy at 5172 correlate with lower mtDNA copy number. On the other hand, the expression of mitochondrial *mt-Co1* positively correlates with 12 A heteroplasmy (*ρ* = 0.5646, *P* = 0.0033, Spearman test, Fig. 2e), while no significant difference is observed between strains (Fig. 2f). Consistent with the latter observation, the levels of several OXPHOS proteins as well as those of the nuclear-encoded beta-actin protein are similar between strains (Fig. 2g,h). These findings suggest that while higher levels of 12 A heteroplasmy in the OriL lead to a reduction of mtDNA copy number, the cells are capable of a compensatory increase in the expression of genes encoded by mtDNA despite its decreased copy number.

### Mitochondrial function in young mice correlates to 12 A heteroplasmy in the OriL when normalized to mtDNA copy number

**Figure 3:**
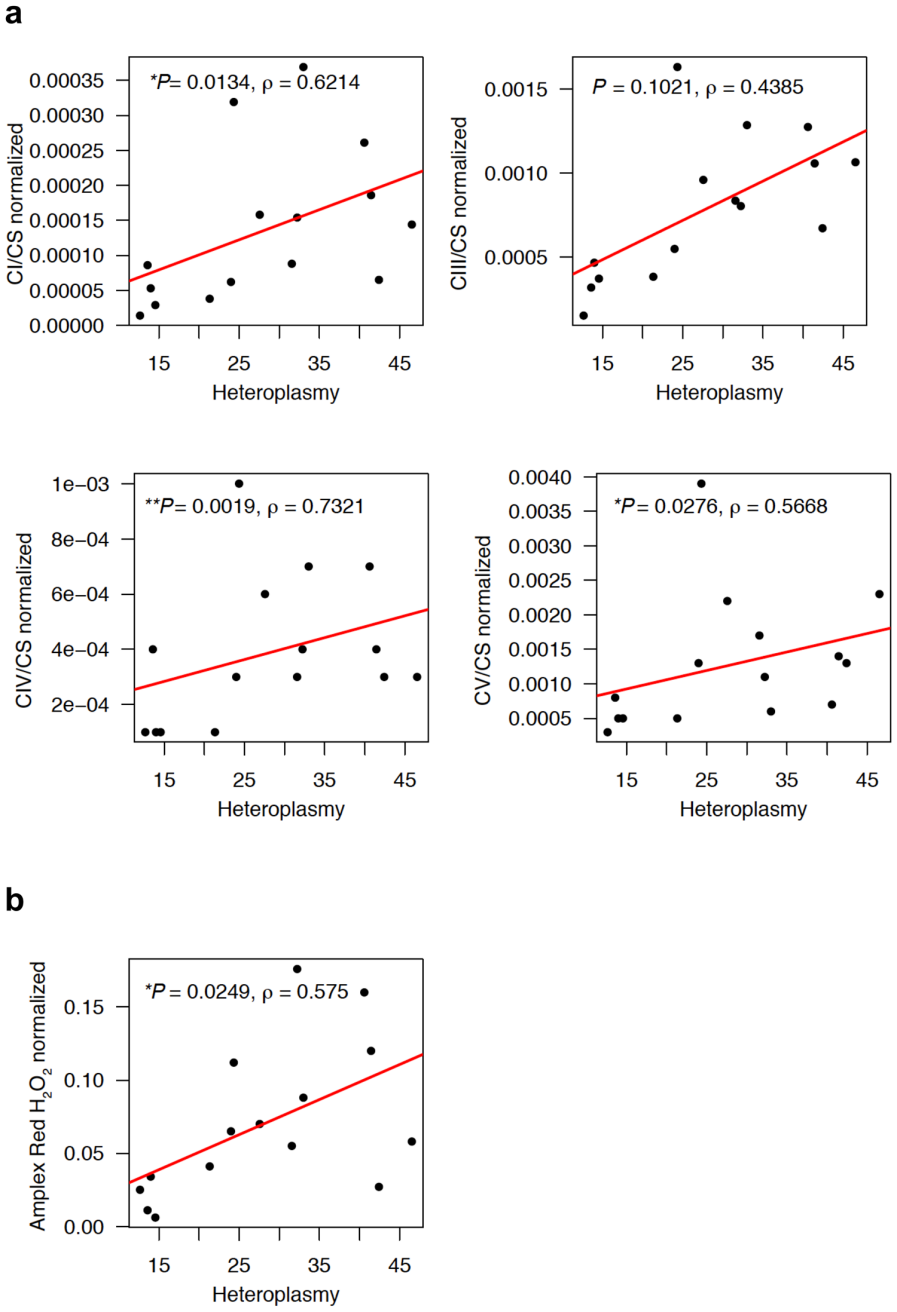
Mitochondrial functions in young mice correlate to the levels of 12 A heteroplasmy at OriL. **(a)** Oxidative phosphorylation (OXPHOS) complex activities measured using liver mitochondria obtained from young mice (three months old, females), normalized to the individual mtDNA copy number ratio (*mt-Co1/Vdac1*). Complex I (CI) activities were normalized to citrate synthase (CS) activities. Complex III (CIII), complex IV (CIV) as well as complex V (CV) values were also analyzed in the same manner. Complex I (CI) activities normalized to citrate synthase (CS) activities significantly correlate with the levels of 12 A heteroplasmy in OriL when normalized to the individual mtDNA copy number ratio. N = 7 (B6), n = 8 (B6-mt^AKR^). Correlations were investigated using the Spearman rank correlation. **(b)** Levels of hydrogen peroxide in female liver mitochondria obtained from young mice (3–5 months old) normalized to the individual mtDNA copy number ratio (*mt-Co1/Vdac1*). The normalized ROS levels show a positive correlation with the levels of 12 A heteroplasmy in OriL (ρ = 0.575, *P* = 0.0249, Spearman test). N = 7 (B6), n = 8 (B6-mt^AKR^).

Mitochondrial functional analysis including OXPHOS complex activity and hydrogen peroxide measurement assays display no significant correlation with 12 A heteroplasmy in the OriL (Supplementary Fig. S2a,b). However, when normalized to the ratio of mtDNA to nDNA copy number, 12 A heteroplasmy significantly correlates with mitochondrial OXPHOS complex I, IV and V activity (the values of each complex activity were normalized to the individual values of citrate synthase (CS); complex I (CI/CS) ρ = 0.6214, *P* = 0.0134; complex IV (CIV/CS) ρ = 0.7321, *P* = 0.0019; and complex V (CV/CS) ρ = 0.5668, *P* = 0.0276, Spearman test, Fig. 3a) as well as ROS levels (ρ = 0.575, *P* = 0.0249, Spearman test, Fig. 3b). This phenomenon is observed only in young mice (three to six months of age; Fig. 3a,b), while an aged population maintains the trend of this correlation (approximately 18–20 months of age; Supplementary Fig. S3a,b).

### Impaired glucose and lipid metabolism may determine the healthspan in B6-mt^AKR^ mice

**Figure 4:**
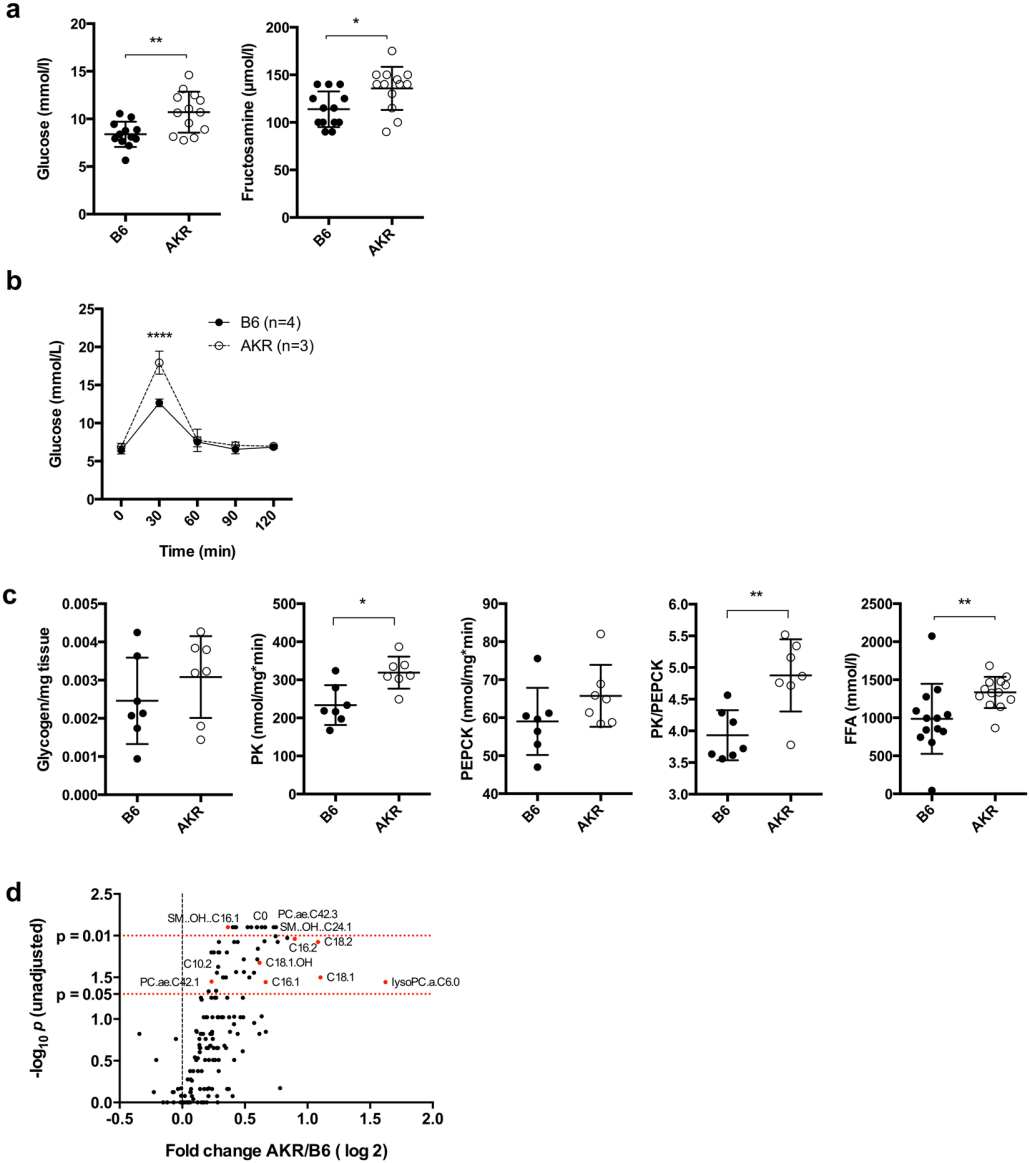
Impaired glucose and lipid metabolism in B6-mt^AKR^ mice. **(a)** Glucose and fructosamine levels were determined in plasma samples from random-fed B6 and B6-mt^AKR^ mice (3 M, females). *P = 0.0106 (fructosamine), **P = 0.0045 (glucose), Mann-Whitney U test. N = 12–13 per strain. AKR = B6-mt^AKR^. **(b)** Oral glucose tolerance test (2 g/kg, p.o.) was performed in young (3 months old) B6 and B6-mt^AKR^ female mice. ****P < *0*.*0001*, two-way ANOVA. **(c)** Glycogen content, activities of pyruvate kinase (PK) and those of phosphoenolpyruvic carboxykinase (PEPCK), and the ratio of PK to PEPCK were determined in liver samples obtained from the mice tested in **a**. Free fatty acids levels were measured in plasma samples obtained from overnight-fasted B6 and B6-mt^AKR^ (3 M, females, same animals tested in **a**). *P = 0.0111 (PK), **P = 0.0041 (PK/PEPCK), **P = 0.0027 (FFA), Mann-Whitney U test. **(d)** Liver lipid metabolites (free carnitine, acylcarnitines, phosphatidylcholines, lysophosphatidylcholines and sphingomyelines) were measured in B6 and B6-mt^AKR^. AKR = B6-mt^AKR^. Females, n = 5/strain.

Mitochondrial metabolism has a well-defined impact on glucose metabolism^24,25^, and conversely, increased glucose levels promote systemic aging^26^. We thus lastly evaluated the putative impact of 12 A heteroplasmy in the OriL on the metabolic phenotype of both B6-mt^AKR^ and B6 mice. Random-fed plasma glucose levels are higher in 12 A heteroplasmy B6-mt^AKR^ mice than in B6 controls (Fig. 4a). Consistently, plasma fructosamine concentrations, which reflect the glucose levels over a 2–3 week period prior to measurement, are also increased in states of high 12 A heteroplasmy (Fig. 4a), indicating a long-term elevation of plasma-glucose in these mice. Moreover, B6-mt^AKR^ consistently display elevated levels in oral glucose tolerance tests (Fig. 4b). Interestingly, no difference was observed in glucose levels upon fasting or in i.p. glucose tolerance tests (Supplementary Fig. S4a). Since insulin levels in both fasted- and random-fed plasma samples are unaltered, as are lactate, cholesterol and fasted glucose levels (Supplementary Fig. S4b,c), these data suggest enhanced intestinal glucose uptake, but no insulin resistance in our study. Furthermore, comparable levels of glycogen storage contrasted by higher activity of pyruvate kinase (PK, indicative for glycolysis) and phosphoenolpyruvate carboxykinase (PEPCK, indicative for gluconeogenesis) (Fig. 4c) indicate an increase in glucose consumption in B6-mt^AKR^ mice. Higher levels of free fatty acids are also present in B6-mt^AKR^ (Fig. 4c), suggesting a global reduction of beta-oxidation in these mice. This is supported by our findings of elevated long-chain fatty acids in liver samples of B6-mt^AKR^ (Fig. 4d and Supplementary Fig. S4d), being consistent with an impaired capacity for mitochondrial metabolism, and in particular beta-oxidation. Thus, mice with higher 12 A heteroplasmy (B6-mt^AKR^) exhibit skewed glucose and lipid metabolism, and that may be considered causal for the impaired lifespan of B6-mt^AKR^ mice in comparison to low-heteroplasmy B6 control mice. This is consistent with long-standing published evidence that mitochondrial mutations, such as the m.3243 A>G mutation that is causal for so-called mitochondrial diabetes mellitus, play a role not only in mitochondrial function, but also in glucose metabolism, as reviewed elsewhere^24,25^.

### The gene expression pattern in mice with high levels of 12 A heteroplasmy at the OriL is similar to that in advanced age mice

**Figure 5:**
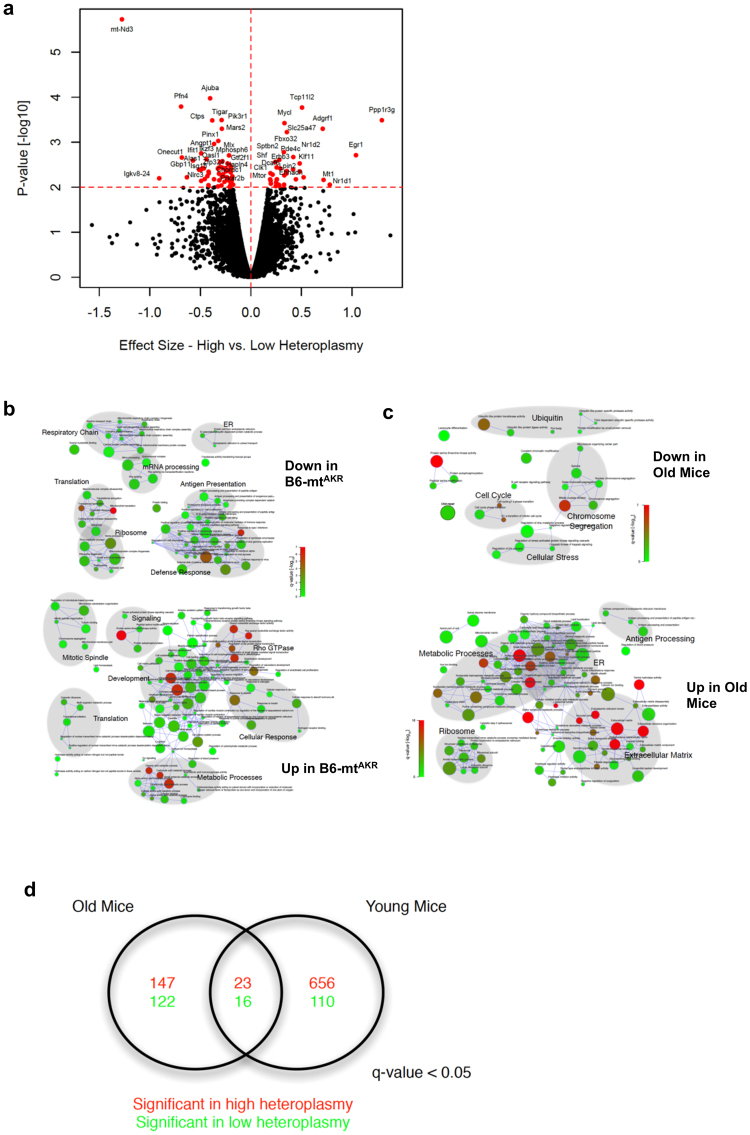
RNA-seq analysis of liver samples of B6-mt^AKR^ and B6 displayed differential gene expression between the strains. **(a**) Differentially expressed genes between B6-mt^AKR^ and B6 mice. The volcano plot demonstrates the effect size versus the −log10 *p-*value of differentially regulated genes. Genes whose expression were *p* < *0*.*01* were plotted in red. All young and aged mice were included in the analysis. **(b**) Gene set enrichment analysis of genes whose expression was affected by the 12 A heteroplasmy levels. The network represents the significant gene sets as nodes; with the node color corresponding to the significance and the node diameter to the gene set size. Nodes are connected by edges if they share at least 20% of their genes. Areas highlighted in gray background indicate the most prominent function in the clustered nodes. **(c**) Gene set enrichment analysis of genes between young and aged mice. The same method as (**b**) was used for data analysis. (**d**) Biological process ontology terms commonly affected by aging and 12 A heteroplasmy levels. Of 23 significantly up-regulated biological processes controlled by both aging and heteroplasmy, metabolic processes were up-regulated in aged mice and mice with higher 12 A heteroplasmy levels. On the other hand, the mitochondrial processes were down-regulated.

Next, to elucidate the pathways involved in heteroplasmy-related phenotypes, RNA-seq was performed on liver tissue from 3–4 and 19–20-month-old B6 and B6-mt^AKR^ mice. Of 14,031 expressed genes, 103 are differentially expressed between young B6-mt^AKR^ and B6 mice (*p* < *0*.*01*, Fig. 5a, Supplementary Data S1). Genes highly expressed in mice with higher heteroplasmy include *Mtor* and *Fbxo32*, suggesting that heteroplasmy exhibits a mild, but significant impact on the mTOR pathway. Interestingly, *Fbxo32* (Atrogin-1; Mafbx) has been shown to be upregulated in sarcopenia. Pathways leading to sarcopenia include the down regulation of the PI3K/AKT pathway and activation of the FOXO transcription factor^27^.

Gene set enrichment analysis (GSEA) reveals that up-regulated genes include those involved in metabolic processes and cellular responses (mainly to hormones), while down-regulated genes include those involved in the respiratory chain, ribosomes and translation (e.g., mitochondrial translation) (Supplementary Data S2, Fig. 5b). We also evaluated whether aging and heteroplasmy play complementary roles in driving gene expression. The overlap between GSEA according to heteroplasmy versus age (Fig. 5c) results in 23 common highly expressed gene ontology (GO) terms and 16 down-regulated terms (Fig. 5d). Glucose metabolism and cell adhesion were prevalent in mice with higher heteroplasmy (B6-mt^AKR^) and aged mice, while mitochondrial processes were prevalent in those with lower heteroplasmy (B6) and young mice (Supplementary Data S3). These transcriptomics data are in agreement with the findings of mtDNA copy number and gene expression (i.e., less mtDNA copy number and higher mtDNA-gene expression in B6-mt^AKR^; Fig. 2a,d,e), as well as the metabolic phenotype data, i.e. genes involved in metabolic processes are up-regulated, presumably in order to control glucose metabolism in mice with higher levels of 12 A heteroplasmy (Fig. 4 and Supplementary Fig. S4).

### The impact of low-level heteroplasmic mutations depends on the location

Lastly, we evaluated whether another heteroplasmic mutation present in the mice has an impact on lifespan. Sequencing data revealed that both the B6 and B6-mt^AKR^ strain carry a heteroplasmic mutation at nt9821, in the tRNA-arginine gene (*mt-Tr*). Almost all B6 samples are 8-adenine repeat (8 A) homoplasmic, while 9 A is the major genotype in B6-mt^AKR^ with a very low copy number of 8 A (Supplementary Fig. S5a). However, the level of heteroplasmy in *mt-Tr* does not correlate to lifespan in our study (Supplementary Fig. S5b), indicating that the impact of lower heteroplasmy on lifespan is highly dependent on the location.

## Discussion

Individual mammalian cells, including oocytes, carry thousands of individual copies of mitochondrial DNA. These copies may differ in regards to individual bases or entire regions. This so-called heteroplasmy of mitochondrial DNA has been described many years ago^28–30^ and is known to cause mitochondrial dysfunction if it affects a high percentage of mtDNA copies, i.e., over 70–80%^31,32^. Here, we observed “low-level” heteroplasmy at nt5172 in the OriL (>20%) in the B6-mt^AKR^ mouse line, which is more pronounced than the degree of heteroplasmy observed in the B6 background (namely, ~10%).

This is the first report demonstrating the impact of naturally occurring low-level heteroplasmic mutations, and specifically in the OriL locus, on mammalian aging. Interestingly, the effect of heteroplasmic mutation in the OriL on the lifespan was limited to females. This is in contrast to the well-described “mother’s curse”, i.e. the consequence of mtDNA mutations are often thought to be higher in males^33^. The “mother’s curse” is defined by the fact that numerous male-harming mutations accumulate in mitochondrial genome when those are neutral, beneficial or only slightly deleterious in their effects in females, i.e. a sex-specific selective sieve^34^. In all our conplastic mouse strains, we do not see any mtDNA mutations that specifically affect males. Similar observations were previously reported by a study in fruit flies^35^. In that study, an *ND2* gene mutation, causing a Leigh syndrome-like neurological phenotype, was observed only in females, and the impact of the mutation on the lifespan was greater in females than males^35^. Another example of a mtDNA mutation with a stronger impact in females was described in our previous study, which showed that females, but not males, carrying a single mutation in complex V gain more weight when fed a western-type diet compared to the wild-type mice^36^.

The OriL is indispensable for mtDNA maintenance in mice^22^. The OriL sequence forms a stable stem-loop structure when mtDNA is single-stranded during replication, and mtDNA-directed RNA polymerase (POLRMT) initiates primer synthesis from the polyA stretch of the loop^37^. Since the region is critical for mtDNA replication and maintenance, the OriL appears to be well protected from the incidence of mutations^22^. Our transcriptome data demonstrates that the lower the level of 12 A heteroplasmy in the OriL, the higher the up-regulation of mitochondrial translation, supporting a functional link between the level of 12 A heteroplasmy in the OriL and mtDNA copy number.

The levels of the 12 A heteroplasmy at the OriL correlate to, mitochondrial functions, i.e., the levels of OXPHOS activities (normalized to each individual CS value) and hydrogen peroxide (H_2_O_2_) levels when values are normalized with the mtDNA/nDNA copy number ratio. This correlation was stronger in young mice than in aged mice. This could be likely because aging differentially affects OXPHOS activities and H_2_O_2_ levels in both strains.

We also observed an impact of the different levels of the heteroplasmic mutation at the OriL on glucose metabolism in mice. To the best our knowledge no variations in OriL have been reported to be associated with mitochondrial diabetes. This is in agreement with previous reports of mtDNA mutations, e.g., the m.3243 A>G mutation in the mitochondrial DNA-encoded tRNA (*Leu*, *UUR*) gene is causal for mitochondrial diabetes^38^. The precise functional consequence of the m.3243 A>G mutation in tRNA-Leu (UUR) on mitochondrial function, leading to mitochondrial diabetes, is a matter of debate, but it is likely to involve 1) alteration in the ATP/ADP ratio, which is a critical determinant of insulin secretion in pancreatic beta cells, 2) functional impairment of the respiratory chain, and 3) an imbalance in the mtDNA-encoded protein amount^24^. Additionally, it was shown that the gene expression patterns in cells carrying the m.3243 A>G mutation were quite similar to those in cells with reduced mtDNA copy numbers, indicating the relevance of the mutation and the mtDNA copy number^24^. Interestingly, experimental evidence demonstrated that the reduced mtDNA copy number induced by beta-cell specific disruption of the nuclear genome-encoded mitochondrial transcription factor A gene (*Tfam*), which controls mtDNA copy number and transcription, impaired glucose-induced insulin responses, consequently resulted in mitochondrial diabetes^39^. Another report showed that mutations at the H-strand replication origins (OriH) of mtDNA are also linked to the levels of the mitochondrial DNA copy number and diabetes^40^. Similarly, mutations in mitochondrially encoded genes, or mitochondrially active nuclear encoded genes, frequently associated with glucose intolerance, cause neurodegenerative disorders^41^. One example of such disorders is Friedreich Ataxia (FA), which is resulted by the impaired expression of the nuclear genome encoded frataxin protein that affect OXPHOS function^42^ by mediating mitochondrial iron-sulphur-cluster biosynthesis^43^. FA patients develop diabetes and exhibited decreased lifespan^41^, and experimental evidence using different models showed that a frataxin knock-out cause diabetes in mice^44^, and knocking down of the frataxin gene resulted in shorter lifespan in worms^45^.

To date, only a few studies have addressed the functional and pathological consequences of mutations in the OriL in humans. One study identified a rare mutation in OriL (m.5747 A>G mutation, i.e., the starting point of poly-A in the loop structure, which is similar to the 5172 mutation in mice) in 2 of 133 bipolar disease patients, while none of 186 healthy control carried the mutation^46^. Another study reported a rare mutation in OriL (m.5752insA, which is in the poly-A loop structure) in one female with hypertension^47^. Since the position is evolutionally conserved^46^, the mutation is likely pathologically relevant, though its relevance to the mtDNA copy number and disease phenotypes have not been fully investigated^48^.

Our findings indicate that the correlation of the higher levels of the 12 A heteroplasmy at the OriL to the mitochondrial DNA copy number and glucose metabolism, may result in a shorter lifespan in mice. As a proof of this concept and to identify the pathways involved, generation of cell lines (eventually mice) with different levels of the heteroplasmy at OriL using gene-editing technology, e.g., mitochondrial genome-target CRISPR-Cas system^49^ or TALEN^50,51^, and an investigation on the aging and metabolic phenotypes of the cells carrying different levels of the heteroplasmy should be performed.

Additionally, this particular region in the OriL seems to be a specific target of the nematodal CLK-1 protein^52^. The same study demonstrated that its mouse homologue also displays binding activity specific to the OriL loop, suggesting a regulatory function of CLK-1 in mtDNA replication in addition to its known ubiquinone biosynthesis function^52^. Mutations in the *clk-1* gene result in an extension of lifespan in nematodes^53^, and a partial inactivation of the mouse homologue *Mclk-1* also extends lifespan in mice^54^. Global deletion of *Mclk-1* in adult mice results in decreased levels of fasting glucose and triglycerides^6^, suggesting the potential role of the OriL in metabolic control. This experimental evidence supports our observation regarding the correlation between heteroplasmic mutations in OriL and lifespan in mice. To determine whether the interaction between CLK-1 and OriL is dependent on the levels of 12 A heteroplasmy at the OriL, experiments such as DNA binding activity of CLK-1 protein using probe containing 11 A or 12 A at 5172 at the OriL as previously described^52^ will be considered. Additionally, since the loss of CLK-1 extends the lifespan in worms and mice, it seems that disrupting of the binding CLK-1 and OriL may negatively regulate of mtDNA replication, which consequently leads to a shorter lifespan. Thus, analyzing the effect of Mclk-1 knockout or overexpression on the B6-mt^AKR^ and B6 backgrounds, may elucidate the impact of the *Mclk-1* gene on different levels of the 12 A heteroplasmy and lifespan *in vivo*.

An additional low-level heteroplasmy at position 9821 is present in our data, in B6-mt^AKR^ mice. This position is located in the tRNA-arginine gene (*mt-Tr*), which is known to be polymorphic in common inbred strains^21,55,56^. No correlation is observed between the heteroplasmic mutation in *mt-T*r and any of the phenotypes we investigated. Interestingly, a previous study investigating the functional consequences of varying length polymorphisms of adenines at nt9821 in the DHU loop of *mt-Tr* reported that cybrids carrying 8 A and 9 A polymorphisms did not differ the levels of H_2_O_2_, and a copy number ratio of mtDNA to nDNA, while those levels were significantly higher in cybrids with 10A^57^. B6-mt^AKR^ mice exhibited low levels of the heteroplasmy, i.e. 8 A or 10 A at 9821 in *mt-Tr*, 5% and 0.56% respectively, while all B6 mice carry homoplasmic 8 A variant. Those very low levels of heteroplasmy are unlikely to cause the observed phenotypes in the B6-mt^AKR^ mice. Also, the 10 A heteroplasmy, which is reportedly a causal variant for OXPHOS functional differences, occurred in only 7 of 32 B6-mt^AKR^ mice, thus, supporting our conclusion that the impact of low-level heteroplasmy is dependent on the location within the mtDNA, i.e. OriL in this study.

In summary, we present experimental evidence linking the degree of mtDNA heteroplasmy to murine lifespan. Specifically, higher levels of 12 A heteroplasmy at position 5172 in the OriL correlate with shorter lifespan and impaired glucose metabolism in female mice. Furthermore, this heteroplasmy influences mtDNA copy number. In young mice, such a reduction of mtDNA copy number can be compensated for by increased expression of the affected genes. In contrast, this compensatory mechanism is missing in aged mice. The heteroplasmic mutation investigated in this study is natural, low-level and stable over generations. We demonstrate that such quiescent variation in mtDNA influences healthspan even under normal housing condition, suggesting that additional environmental triggers such as metabolic stress could amplify the phenotypic consequences. Further studies to elucidate the involved mechanisms, particularly the interaction between mtDNA variants and environmental stress factors, are warranted. Given the established role of mtDNA variations in human exercise performance, the current findings may likely translate into a co-determining function of human aging.

## Methods

### Mice

#### Animals and husbandry

The conplastic mouse strain *C57BL/6J-mt*^*AKR/J*^ (B6-mt^AKR^) was previously generated^20^. *C57BL/6 J* (B6) mice (stock number 000664) were purchased from Jackson laboratory (Bar Harbor, ME, USA) and were inbred, supplying new breeders every 12 months. B6-mt^AKR^ mice with a backcross of 25 to 28 generations were used for the survival study. Nuclear genomes of both B6 and B6-mt^AKR^ were genotyped using the MegaMUGA Mouse Universal Genotyping Array (77,800 SNPs) as described below, and greater than 99.9% of SNPs were identical between the strains (Supplementary Table S2). The entire mitochondrial genome was deep-sequenced using Illumina MiSeq as described below.

Mice had *ad libitum* access to filtered water and autoclaved pellet diet (1314, Altromin, Lippe, Germany). The animal facility was maintained at 21 °C on a 12-h light-dark cycle. Mice were allocated into two study groups: a longitudinal study group to evaluate lifespan and a cross sectional study group to evaluate the mice at different ages. Animal use and all methods were approved by the Animal Care and Use Committee (V242–7224. 122–5, Kiel, Germany), and were performed in accordance with the relevant guidelines and regulations by certified personnel.

#### Next generation sequencing of the entire mitochondrial genome

Total DNA was isolated from liver, heart and brain tissues from mice at 3 and 22 months of age and was obtained using the DNeasy Blood and Tissue kit (Qiagen, Hilden, Germany). Two primer sets [6551 forward (AGCAAAAGCCCACTTCGCCA) and 15169 reverse (GGTTGGCCCCCAATTCAGGT); 15150 forward (ACCTGAATTGGGGGCCAACC) and 6573 reverse (TGGCGAAGTGGGCTTTTGCT)] were used to amplify the mtDNA by long-range PCR^58^. The 15169 reverse primer and the 15150 forward primer overlap. The library preparation was performed using a Nextera XT DNA Library Preparation Kit (Illumina Inc., CA, USA), and the 10 pM library was sequenced using the Illumina MiSeq sequencing platform (2 × 150 bp) (Illumina Inc.).

Trimmed paired-end fastq files were obtained from MiSeq platform. Quality of the paired-end reads was evaluated using the FastQC v0.11.5 software^59^. Reads missing either ends or with an average quality score of <30 or a read length of <30 bp were discarded from subsequent analysis. The remaining reads were mapped to the C57BL/6 J mouse mtDNA reference sequence (EF08336) using Burrows-Wheeler Aligner bwa version 0.705^60^, and bam files were generated. Duplicated reads generated due to PCR were removed using Markdupliates (Picard tools version 1.92^61^), and indels were realigned using IndelRealigner (Genome analysis tool kit version 3.3)^62^. The processed bam files were assessed for the frequency and base quality (≥30) for each reference and alternate base in mouse mtDNA using pysamstat (version 0.24.3)^63^. When the frequency of the alternate allele compared with the reference allele was greater than 90%, it was considered as homoplasmic mutation, whereas 40 to 90% was considered as heteroplasmy. Additionally, bam files were manually inspected for mutations and indels using IGV software^64^.

To count the GA+ at nucleotide 5171 and 5172 and the TA+ at nucleotide 9821, the CIGAR strings from the bam files were parsed using an in-house-written perl script. In brief, samtools were used to identify the mapped paired-end read at this position. Thereafter, these reads were search for motif G+ poly A (reference, 11A) and occurrence of every motif was counted in each sample. The quality of each motif was also parsed, where average base pair quality <20 was discarded. To the targeted re-sequencing the above procedure was applied as same, while aligning the reads to the targeted region spanned by the primer sequences.

#### Nuclear genome SNP genotyping

DNA samples were genotyped for 77,808 SNP markers in the nuclear genome using the Mouse Universal Genotyping Array (MegaMUGA, GeneSeek Europe, Scotland, UK). Array processing and genotype calling were performed using GeneSeek as previously described^65^.

#### Whole transcriptome study

Total RNA was prepared from liver tissue using innuPREP RNA Mini Kit (Analytik Jena AG, Jena, Germany). Library preparation was performed employing the TruSeq stranded Total RNA kit (Illumina Inc.), starting from 0.6 µg of total RNA of each sample, and sequenced on an Illumina HiSeq. 4000 platform (75-nucleotide paired-end reads). Aiming for high transcriptome coverage, sequencing runs were performed by combining four samples per lane (corresponding to approximately 50 million reads per sample). Fastq reads were pseudo-aligned to the mm10 genome assembly using Kallisto^66^ and transcript read counts were aggregated to Ensembl Gene IDs for further analysis. RNAseq data has been uploaded to Gene Expression Omnibus (GEO) with the ID GSE94315. The reviewer can access the data under the private link: https://www.ncbi.nlm.nih.gov/geo/query/acc.cgi?token=orcniykedvejjox&acc=GSE94315. Differential gene expression analysis was performed via the R library sleuth^67^ using a linear model that accounted for mouse age, heteroplasmy and batch effects. Significance and effect sizes of differential gene regulation were calculated from the likelihood ratio and the Wald test, respectively, as implemented in the sleuth package. GO term and pathway enrichment analyses were performed based on the effect size between the different mouse strains or age using the generally applicable Gen Set Enrichment Analysis (GSEA), GAGE, which determines whether a set of genes is systematically up- or down-regulated as a whole^68^. For gene set definitions, we used the Molecular Signatures Database (MSigDB) collections v.5.2 that were mapped to mouse ortholog Entrez IDs via the HGNC Comparison of Orthology Predictions (HCOP) (provided by the Walter and Eliza Hall Institute; http://bioinf.wehi.edu.au/software/MSigDB/). Gene sets with less than 10 or those with more than 500 members were discarded for statistical robustness and biological interpretation. To assess the significance of differential regulation, a gene rank-based nonparametric Kolmogorov-Smirnoff test was used.

### Survival study

#### Lifespan study and determining age at death

Sixty-four mice per group were calculated using G*Power^69^ to allow the detection of a 10% difference in lifespan at a two-sided 0.05 significance level and a power of 0.8. Therefore, we used 155 B6 mice (87 females and 68 males) and 131 B6-mt^AKR^ mice (85 females and 79 males) to evaluate lifespan. Mice were inspected daily. As previously described^70^, a mouse was determined as moribund when demonstrating more than one of the following clinical signs: inability to eat or drink; abnormally low body temperature; severe lethargy (reluctant to move when gently prodded with forceps); severe balance or gait disturbance; rapid body mass loss; an ulcerated or bleeding tumor; enlarged abdomen; panting; or severe ulcerative dermatitis that covers greater than 20% of the body surface. The age at which a moribund mouse was sacrificed was taken as the best available estimate of its natural lifespan. At the autopsy, the aging phenotype (presence of alopecia, graying hair, body mass reduction, and kyphosis) was scored as previously described^23^. Moribund mice were autopsied by a veterinarian, and clinical and macroscopic observations were recorded. Blood samples were stored at −20 °C, and organ samples were stored in 4% paraformaldehyde solution in phosphate buffered saline.

#### Statistical analysis for the survival study

Survival curves were drawn using the Kaplan-Meier method, and median lifespans with 95% confidence intervals were calculated. Censored mice are displayed as a cross (×) in the survival curves. The differences of longevity between the strains were analyzed using both the log-rank method and the Gehan test. In addition, mouse survival data in females were fitted by a skew Gaussian distribution using the R library sn0^71^.

### Mitochondrial functional studies

#### Mitochondrial DNA copy number ratio

The copy number ratio of mitochondrial DNA and nuclear DNA was determined using a droplet digital PCR system with DNA-binding dye (Bio-Rad Laboratories, Munich, Germany). Genomic DNA isolated from liver samples was digested with HindIII (New England BioLabs Inc. MA, USA). Droplets were generated using 20 µl PCR reaction components (digested genomic DNA, *mt-Co1* [forward (ACATGAAACCCCCAGCCATA) and reverse (TTGTGTTTAGGTTGCGGTCTG)], *mt-Nd5* [forward (TGCCTAGTAATCGGAAGCCTCGC and reverse (TCAGGCGTTGGTGTTGCAGG)] or *Vdac1* [forward (CTCCCACATACGCCGATCTT) and reverse (GCCGTAGCCCTTGGTGAAG)] primers at a final concentration of 1 nM, and 10 µl of 2 × QX200 EvaGreen ddPCR supermix (Bio-Rad Laboratories)) with 70 µl of Droplet Generator Oil for EvaGreen (Bio-Rad Laboratories) using QX200 droplet generator (Bio-Rad Laboratories), followed by PCR reaction using thermal cycler (Bioer, Zhejiang, China). The PCR reaction and the droplet quantification were performed as previously described^72^.

#### Mitochondrial gene expression

The mitochondrial gene expression was determined using a droplet digital PCR system. Total RNA isolated from liver samples were prepared for cDNA using First Strand cDNA Synthesis Kit (Thermo Fischer Scientific, Pinneberg, Germany). The following procedure is the same as the method for mitochondrial DNA copy number ratio determination.

#### Mitochondrial preparation

Freshly removed liver was minced and homogenized on ice in ice-cold isolation buffer [0.2 mM EDTA, 250 mM sucrose, 10 mM Tris-HCl pH 7.8, and protease and phosphatase inhibitor (Thermo Fisher Scientific)] using a Potter-Elvehjem glass homogenizer. The liver homogenates were centrifuged at 1000 × *g* for 10 minutes at +4 °C, and the resulting supernatants were centrifuged for 10,000 × *g* for 15 minutes at +4 °C. Pellets were resuspended in a small volume of isolation buffer and immediately used for the measurement of mitochondrial hydrogen peroxide. For the mitochondrial OXPHOS complex activity measurement, mitochondria samples were frozen at −80 °C until analysis.

#### Mitochondrial respiratory chain complex activity assay

Mitochondrial respiratory chain complex activities were measured as previously reported^73,74^ with slight modifications^15^. In brief, assays were performed in 200 µl/well in a 96-well plate at 37 °C (except citrate synthase, which was performed at 30 °C) using a Tecan Infinite 200 PRO series spectrophotometer (Tecan Group Ltd., Crailsheim, Germany). Frozen mitochondria samples were disrupted by two cycles of freezing in liquid nitrogen and thawing at room temperature. Complex I activity was measured at 340 nm as the rotenone-sensitive rate of NADH oxidation and complex I-dependent electron transfer to decylubiquinone. Complex III activity was assayed at 550 nm as the antimycin A-sensitive rate of reduction of cytochrome c by electron transfer from decylubiquinol through complex III. Complex IV activity was measured at 550 nm as the cyanide-sensitive oxidation of cytochrome c. Complex V activity was assayed at 340 nm as oligomycin-sensitive ATP hydrolysis by complex V coupled to pyruvate kinase and lactate dehydrogenase activities and the corresponding oxidation of NADH. Citrate synthase activity was measured as the condensation reaction of oxaloacetic acid and acetyl-CoA to citrate and the subsequent reduction of DTNB (5,5′-dithiobis (2-nitrobenzoic acid)) by coenzyme A as detected at 412 nm.

#### Measurement of mitochondrial hydrogen peroxide

Hydrogen peroxide content in liver mitochondria and skin fibroblast cell lines was assessed using the Amplex Red H_2_O_2_ kit (Thermo Fisher Scientific) following the manufacturer’s protocol.

#### Western blotting

Frozen liver tissue was lysed in RIPA buffer and the supernatant was used for Western blotting. Antibodies against OXPHOS proteins (MitoProfile Total OXPHOS Rodent WB Antibody Cocktail; Abcam, Cambridge, UK) and beta-actin (Cell Signaling Technology), as well as secondary antibodies (polyclonal goat anti-rabbit immunoglobulins-HRP and polyclonal rabbit anti-mouse immunoglobulins-HRP; both from Dako, Glostrup, Denmark) were used. Antibody binding was visualized using the Clarity Western ECL Substrate (Bio-Rad Laboratories) to detect HRP-induced luminescence, Signals were detected using a CCD camera documentation system (FUSION Fx7, Vilber Lourmat Deutschland GmbH, Eberhardzell, Germany) applying variable exposure times. Images were evaluated using ImageJ v1.50b.

### Metabolic phenotyping

#### Oral glucose tolerance test (OGTT)

Female mice were fasted for 6 hours and then glucose (2 g/kg) was orally administrated. Glucose was determined from a tail vein blood before and 30, 60 and 120 min after glucose administration using a glucometer (ACCU-CHEK^**®**^ Performa, Roche Diagnositcs GmbH, Mannheim, Germany).

#### Plasma parameter assay

Determination of glucose, fructosamine, lactate, free fatty acids and total cholesterol in plasma was performed using an automated analyzer (Cobas Mira S, Hoffmann-La Roche, Basel, Switzerland) with the appropriate commercially available reagent kits (glucose HK, fructosamine, lactate FS, cholesterol PAP, Diatools, Villmergen, Switzerland; and NEFA HR, Wako, Neuss, Germany. Plasma insulin levels were measured using an immunoassay-based Mouse/Rat Insulin Kit on a Sector Imager (Meso Scale Discovery, Gaithersburg, MD) according to the manufacturer’s instructions.

#### Enzyme activity and glycogen content determination

Activities of pyruvate kinase (PK) and phosphoenolpyruvate carboxylkinase (PEPCK) as well as glycogen content in the liver were determined as previously described^75^. In brief, PEPCK activity was determined using cytosolic fraction of frozen liver tissue homogenates (female, n = 7 per strain) in a sample buffer containing 65 mM Tris (pH8.0), 6 mM MgCl_2_, 15 µM MnCl_2_. 0.875 mg/ml BSA. 4.65 mM ATP and ADP, 0.6 mM reduced nicotinamide adenine dinucleotide, 5U/ml PK and lactate dehydeogenase. Oxyloacetate was added as substrate in a final concentration of 0.5 mM, and the conversion of reduced nicotinamide adenine dinucleotide was monitored at 340 nm. Sample without substrate and samples with water were used to determine background activity. The same cytosolic fractions and sample buffers, and substrates without MnCl_2_, ATP and PK, and 0.5 mM phosphoenolpyruvate were used to measure PK activity. Glycogen content in the liver was precipitated homogenate using 5% trichloroacetic acid, and dissolved in Lugol´s reagent (Sigma-Aldrich GmbH, Munich, Germany) in 25% (wt/vol) potassium chloride containing 30 mm hydrochloric acid, followed by the measurement at 600 nm and normalized to the tissue weight.

#### Metabolomics study

Plasma and liver samples were obtained from B6 and B6-mt^AKR^ mice (n = 5 per strain; female). Snap frozen samples were sent to Helmholz Center Munich for the targeted metabolites detection (163 metabolites) using Biocrate assays (Biocrates life Sciences AG, Innsbruck, Austria).

#### Statistical analysis

Statistical tests used for analysis are indicated in the figure legends. P-values were not adjusted for multiple testing.

### Wild house mouse samples

217 mice were captured in 34 farms around Espelette, France in September 2013, of which 216 were confirmed to belong to *Mus musculus domesticus*. The sampling procedure was organized over a two days period: the traps were placed at day 1 and trapped live mice were collected in the morning of day 2. Mice were sacrificed and ear samples for analysis were collected and frozen at −20 °C. After shipment of the samples to the laboratory, genomic DNA was extracted from the ear samples using the DNeasy Blood & Tissue Kit (Qiagen) and used for the next generation sequencing of the whole mitochondrial genome, as described for the laboratory mice. Samples from 215 mice were successfully sequenced and used for the analysis.

### Data availability

RNAseq data has been uploaded to Gene Expression Omnibus (GEO) with the ID GSE94315.

## Electronic supplementary material

Supplementary Information

Data S1

Data S2

Data S3
