# Supplementary material for: Low-level mitochondrial heteroplasmy modulates DNA replication, glucose metabolism and lifespan in mice
Source: Sci Rep. 2018 Apr 12;8:5872. doi: 10.1038/s41598-018-24290-6 (PMC5897405; doi:10.1038/s41598-018-24290-6)
Supplement: Supplementary file 1 — Supplementary Information [file 41598_2018_24290_MOESM1_ESM.pdf]

Supplementary information

Low-level mitochondrial heteroplasmy modulates DNA replication, glucose metabolism and lifespan in mice

Misa Hirose, Paul Schilf, Yask Gupta, Kim Zarse, Axel Künstner, Anke Fähnrich, Hauke Busch, Junping Yin, Marvin N Wright, Andreas Ziegler, Marie Vallier, Meriem Belheouane, John F Baines, Diethard Tautz, Kornelia Johann, Rebecca Oelkrug, Jens Mittag, Hendrik Lehnert, Alaa Othman, Olaf Jöhren, Markus Schwaninger, Cornelia Prehn, Jerzy Adamski, Kensuke Shima, Jan Rupp, Robert Häsler, Georg Fuellen, Rüdiger Köhling, Michael Ristow, Saleh M Ibrahim

Figure S1: Analysis for the lifespan in females, excluded earlier death. No difference in aging score and age-related diseases incidence between B6 and B6-mt^{AKR}.

Figure S2: Mitochondrial functions normalized with protein amount do not correlate to the levels of the 12A heteroplasmy.

Figure S3: Mitochondrial functions in aged mice maintain a trend of positive correlation with the levels of the 12A heteroplasmy.

Figure S4: Impaired metabolic phenotypes in B6-mt^{AKR} mice.

Figure S5: Heteroplasmy in *mt-Tr* does not correlate with lifespan in mice.

Table S1: The list of mtDNA mutation in B6 and B6-mt^{AKR} mice.

Table S2: The statistical analysis for the lifespan of B6 and B6-mt^{AKR} mice.

Table S3: Nuclear genome difference between B6 and B6-mt^{AKR} mice.

Data S1: List of differentially expressed genes between mice with higher 12A heteroplasmy levels (B6-mt^{AKR}) and those with lower 12A heteroplasmy levels (B6).

Data S2: List of Biological Process Ontology terms significantly up-regulated or down-regulated in mice with higher 12A heteroplasmy levels (B6-mt^{AKR}) compared to those with lower 12A heteroplasmy levels (B6).

Data S3: List of Biological Process Ontology terms that were commonly affected by aging and the levels of 12A heteroplasmy.

Figure S1

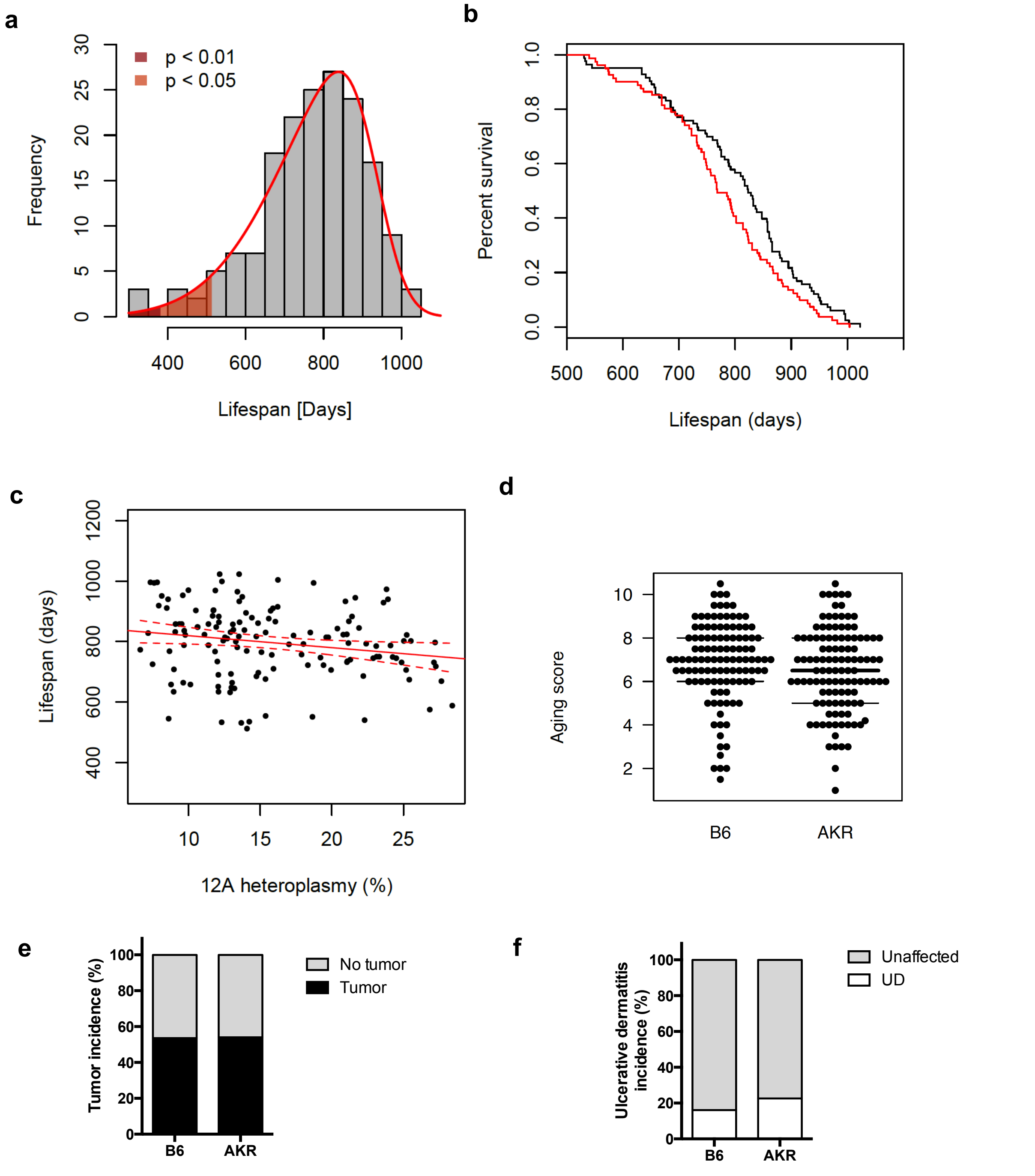

Figure S1: Analysis for the lifespan in females, excluded earlier death. No difference in aging score and age-related diseases incidence between B6 and B6-mt^{AKR}.

(a) Lifespan distribution in females. Lifespan data of all females (both B6 and B6-mt^{AKR}) were fitted to a skew normal distribution. The *P*<0.05 cut-off lies at 517 days.

(b) Using this criterion the deaths before 517 days were eliminated, and survival curves are plotted for female mice. *P*=0.0284, log-rank test.

(c) Correlation between 12A heteroplasmy levels and lifespan of female mice, excluding the deaths before 517 days. ρ= -0.2272, *P*=0.0072, Spearman test. Dashed lines indicate the 95% confidence interval of a linear fit indicated by the solid line.

(d) Aging score was determined in B6 (male, n=47; female, n=67) and B6-mt^{AKR} (male, n=54; female, n=59). The upper and bottom dot lines indicate the 75% and 25% quintile, respectively, while the middle bar shows the median.

(e) Tumor incidence in B6 (male, n=49; female, n=69) and B6-mt^{AKR} (male, n=65; female, n=74).

(f) Incidence of ulcerative dermatitis in B6 (male, n=68; female, n=87) and B6-mt^{AKR} (male, n=78; female, n=84). AKR=B6-mt^{AKR}.

Figure S2

a

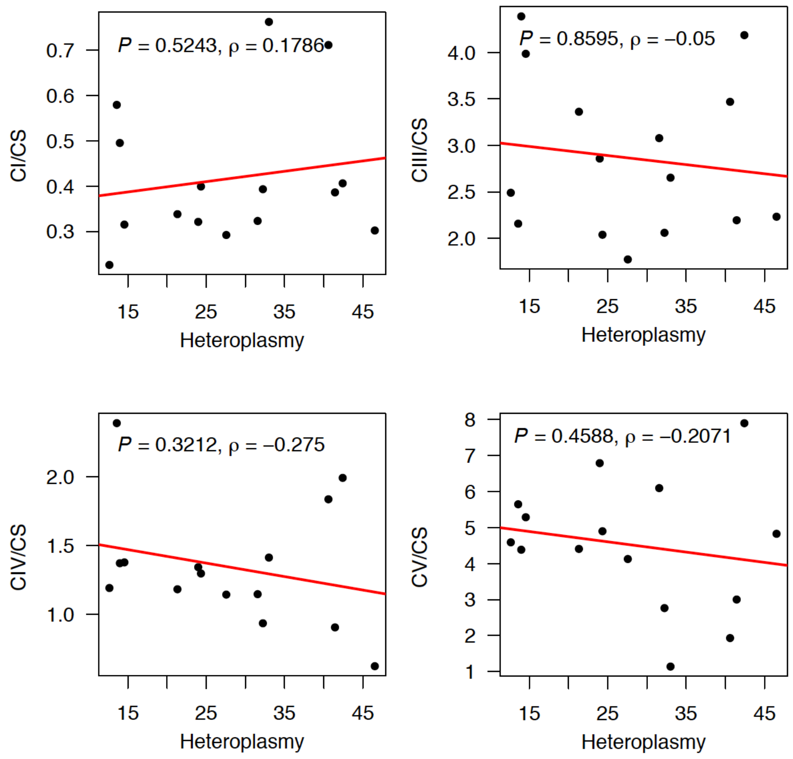

b

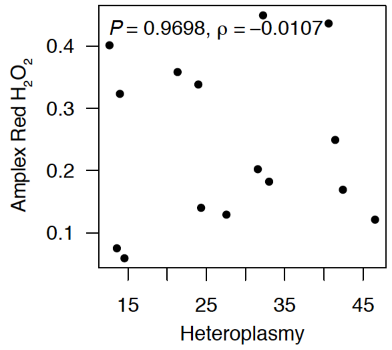

Figure S2: Mitochondrial functions normalized with protein amount do not correlate to the levels of the 12A heteroplasmy.

(a) Oxidative phosphorylation (OXPHOS) complex activities measured using liver mitochondria obtained from young mice (3 months old, female, same values measured in **Figure 3a**) normalized to individual citrate synthase activities. The normalized values do not show a positive correlation with the levels of the 12A heteroplasmy (Spearman rank correlation).

(b) The levels of hydrogen peroxide in liver mitochondria isolated from young mice (3-5 months old, female, same values measured in **Figure 3b**) normalized to protein amount. The normalized ROS values do not show a positive correlation with the levels of the 12A heteroplasmy in OriL (Spearman rank correlation).

Figure S3

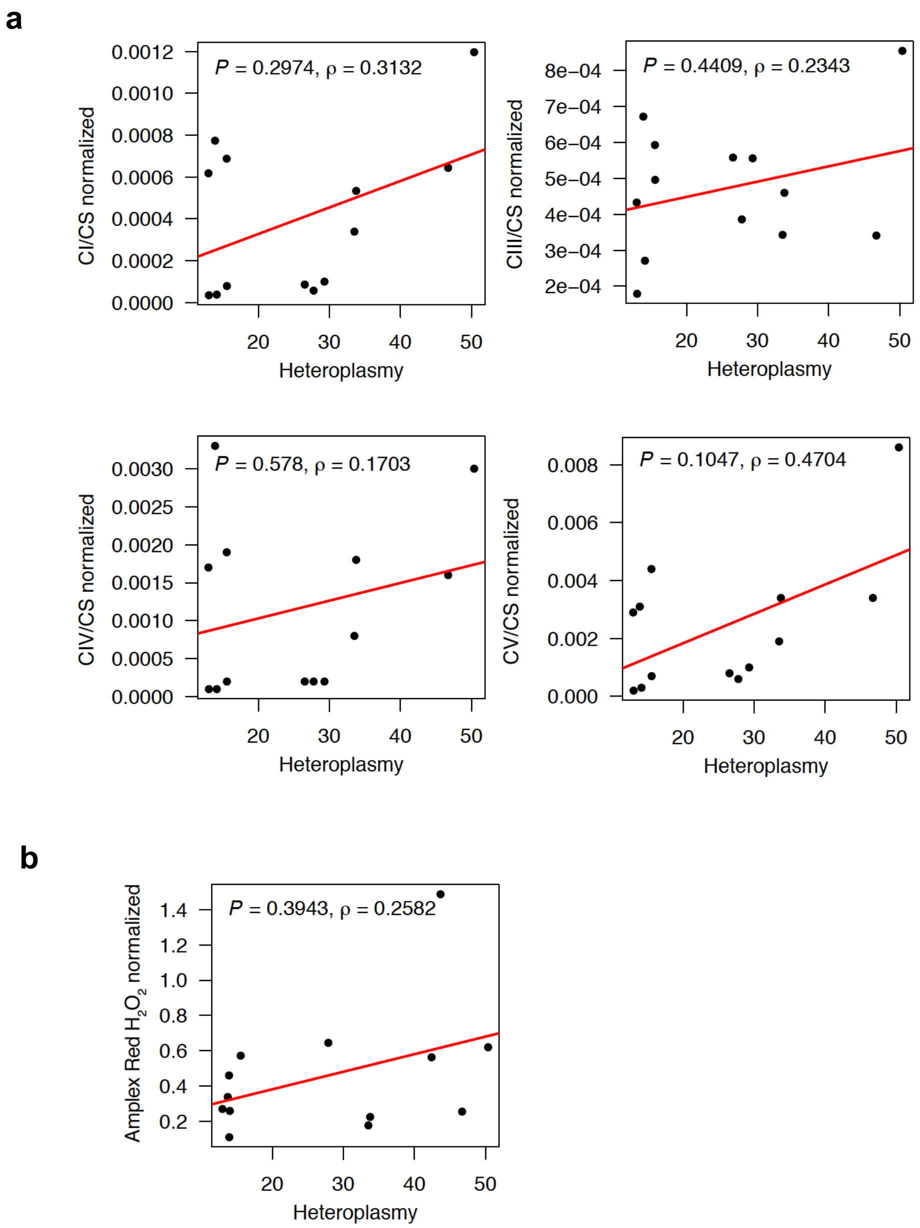

Figure S3: Mitochondrial functions in aged mice maintain a trend of positive correlation with the levels of the 12A heteroplasmy.

(a) OXPHOS complex activities (normalized with the individual activities of citrate synthase) measured in liver mitochondria from aged mice (19-22 months old, females) normalized to individual mtDNA copy number ratio. The trend of the positive correlation between the 12A heteroplasmy levels and the normalized OXPHOS activities remains in aged mice. N=6 (B6), n=7 (B6-mt^{AKR}) (Spearman rank correlation).

(b) Levels of hydrogen peroxide in female liver mitochondria from aged mice (18-22 months old) normalized to the individual mtDNA copy number ratio. The trend of the positive correlation

between the ROS levels and the levels of the 12A heteroplasmy remains in aged mice. N=7 (B6), n=8 (B6-mt^{AKR}) (Spearman rank correlation).

Figure S4

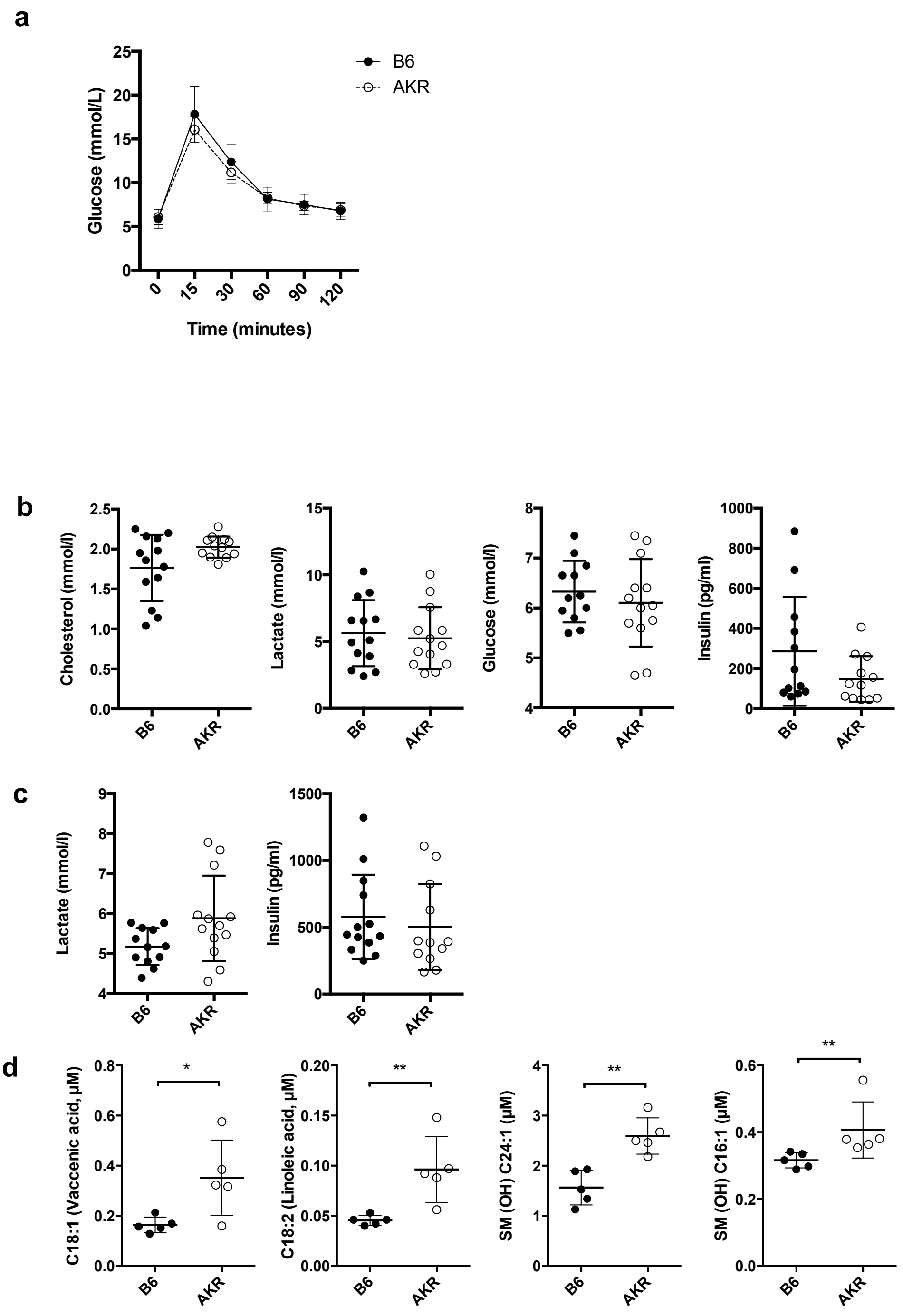

Figure S4: Impaired metabolic phenotypes in B6-mt^{AKR} mice.

(a) Intraperitoneal glucose tolerance test (2g/kg, i.p.) was performed in young (3 months old) B6 and B6-mt^{AKR} female mice. N=7/strain.

(b) Lipid, glucose, lactate and insulin levels were measured in plasma samples obtained from overnight-fasted B6 and B6-mt^{AKR} (3M, females). N=12-13 per strain. AKR=B6-mt^{AKR}.

(c) Lactate and insulin levels were measured in plasma samples from random-fed B6 and B6-mt^{AKR} (3M, females). N=12-13 per strain. AKR=B6-mt^{AKR}.

(d) Representative lipid metabolites measured in liver samples obtained from B6 and B6-mt^{AKR} (female, n=5/strain). **P*=0.0317 (C18:1), ***P*=0.0079 (C18:2, SM (OH) C24:1 and SM (OH) C16:1), Mann-Whitney U test.

Figure S5

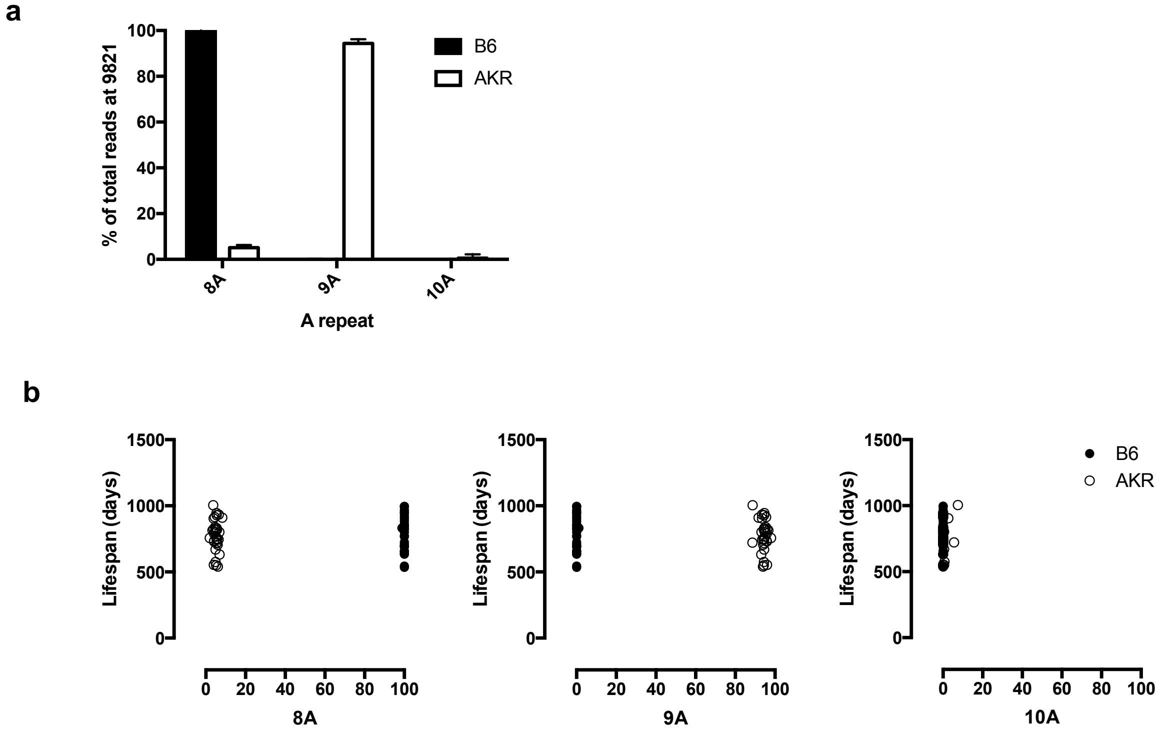

Figure S5: Heteroplasmy in *mt-Tr* does not correlate with lifespan in mice.

(a) Heteroplasmy at 9821 in mitochondrial tRNA arginine gene (*mt-Tr*) is observed in B6-mt^{AKR}. All 32 B6 mice exhibited 8A homoplasmy, while B6-mt^{AKR} carry 8A (32 mice), 9A (32 mice) and 10A (7 in 32 mice) with the levels of 5.10±1.22%, 94.34±1.90% and 0.56±1.66%, respectively (average±SD). AKR=B6-mt^{AKR}, n=32 per strain.

(b) No correlation between heteroplasmy and lifespan is observed.

Table S1: List of mtDNA variations in B6-mt^{AKR} and B6.

Gene	*OriL**	*mt-Nd3*	*mt-Tr*
Strain\ position	5172	9461	9821
B6-mt^{AKR}	*12A, ≥20%*	C	9A
B6	*12A, <20%*	T	8A
AA change	-	Met-Met	-

*The levels of the heteroplasmic mutation were identified using NGS in this study.

Table S2: The statistical analysis for the lifespan of B6 and B6-mt^{AKR} mice.

a

	Log-rank	Gehan
Total	0.4450	0.1014
Female	0.0305	0.0621
Male	0.7075	0.5098

b

	N	Events	Median	95% confidence interval
Female	172	169	792	768-817
Male	147	146	863	842-887

c

	N	Events	Median	95% confidence interval
B6	155	151	841	823-864
B6-mt^{AKR}	164	164	810	791-830

d

		N	Events	Median	95% confidence interval
B6	Female	87	84	817	781-848
	Male	68	67	871	842-904
B6-mt^{AKR}	Female	85	85	767	745-802
	Male	79	79	847	829-890

(a) Statistical analysis for survival curves; B6 versus B6-mt^{AKR}.

(b) Median survival times by sex.

(c) Median survival times by strain.

(d) Median survival times by sex and strain.

Table S3: Nuclear genome difference between B6 and B6-mt^{AKR} mice.

Strain			B6			B6-mt^{AKR}					
Sex			M	F	F	M	F	M	F	F	M
Mouse ID			70015	70012	70004	34596	34580	39350	31977	31991	31976
ChrB37	PosB37	SNPs									
1	42424440	B6_rs31362610	TT	TT	TT	TT	TT	TT	*TC*	TT	*TC*
1	156715218	UNC010465120	TT	TT	TT	TT	*N.D.**	TT	TT	*TG*	*TG*
3	84097284	UNC030314030	GG	GG	GG	GG	*GA*	GG	GG	GG	GG
3	120369799	B6_03-120369799-S	AA	AA	AA	AA	AA	AA	*AG*	*AG*	*AG*
3	121531310	UNC030194728	CC	CC	CC	CC	CC	CC	*CT*	*CT*	*CT*
7	78961795	B6_rs32060039	CC	CC	CC	CC	*CG*	CC	CC	CC	CC
8	26496123	B6_rs33539160	AA	AA	AA	AA	*AG*	*AG*	*AG*	*AG*	*AG*
8	77477256	B6_rs32729089	TT	TT	TT	TT	*TA*	TT	*TA*	*TA*	*TA*
8	120161891	B6_rs32661424	CC	CC	CC	CC	CC	CC	*CT*	CC	*CT*
9	6238770	B6_09-006238770-S	AA	AA	AA	AG	AA	*AG*	AA	AA	AA
10	56034586	B6_rs29377979	GG	GG	GG	GG	GG	GG	*GA*	*GA*	*GA*
10	79915030	B6_rs29349055	AA	AA	AA	AA	*AG*	AA	*AG*	*AG*	*AG*
10	116077282	UNC100129834	TT	TT	TT	TT	TT	TT	TT	*TC*	TT
10	121868277	B6_rs29348001	AA	AA	AA	AA	AA	AA	AA	*AG*	AA
11	87932613	UNC20071212	CC	CC	CC	CC	CC	CC	*N.D.**	CC	CC
14	22151051	B6_rs31151615	TT	TT	TT	TT	TT	TT	TT	*TC*	TT
14	22662231	UNC140101805	AA	AA	AA	AA	AA	AA	AA	*AC*	AA
17	60378784	B6_rs33169019	AA	AA	AA	*AG*	AA	AA	*AG*	AA	*AG*
18	15323094	UNC180052085	AA	AA	AA	AA	AA	AA	AA	*AG*	AA
18	15408257	B6_rs13483221	CC	CC	CC	CC	CC	CC	CC	*CT*	CC
nDNA homology to C57BL/6J (%)			100	100	100	99.997	99.992	99.997	99.987	99.983	99.987

*N.D.; no data due to genotyping errors.
